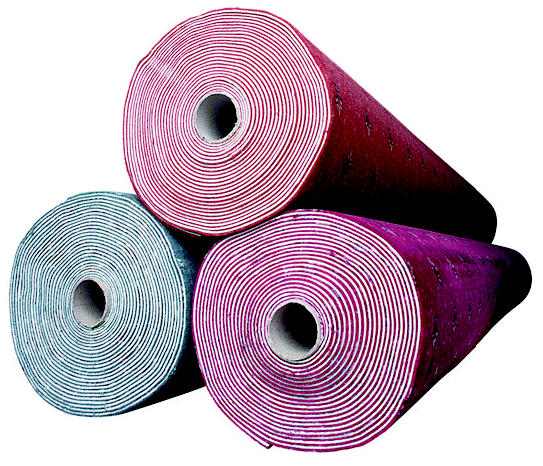# The Beat

**Published:** 2005-12

**Authors:** Erin E. Dooley

## Fly the Environmentally Friendly Skies

In June 2005, the British airline industry unveiled a 15-year initiative to make itself more environmentally friendly. The industry wants to improve its fuel efficiency, reduce perceived external noise, and lower carbon dioxide emissions on new planes by 50% and nitrogen oxide emissions by 80%. Also planned are ways to give travelers information on the amount of fuel used and pollutants emitted on routes that they travel. The industry may also prohibit foreign carriers from flying older, more-polluting aircraft into the United Kingdom.

## A Loan for Colombia

In June 2005 the World Bank announced it was granting a $150 million loan to Colombia to help that nation integrate sustainability principles into its environmental programs and policies and meet the UN Millennium Development Goals, including halving the number of people without adequate water and sanitation facilities. The monies are earmarked for three areas: development of a framework for planning and monitoring the progress toward meeting the UN goals; increased interinstitutional cooperation and public participation in environmental decision making; and development of laws and policies related to air and water quality, solid waste management, and environmental licensing. Bank officials hope the work financed by the loan will also decrease child mortality rates related to respiratory and diarrheal diseases.

## Wave Power in the Works

Just off the northern coast of Portugal is the site of the world’s first commercial wave-generated electric plant. The contract was signed in May 2005 for the $9.6 million project, under which three wave energy converters will be built at the site. The long, hinged converters move with the flow of tidal currents, pumping fluid to hydraulic motors that drive generators.

The wave power plant is expected to provide electricity for more than 1,500 Portuguese households while displacing more than 6,000 metric tons of carbon dioxide produced each year by conventional power plants. If this first phase proves successful, 30 additional wave converters will be ordered by the end of 2006.

## Baytril Gets the Boot from Bird Farms

Amidst calls from doctors and public health advocates, the FDA has banned the use of the antibiotic Baytril in poultry. The FDA is also reviewing requests to ban the use of other drugs given to animals. Although Perdue Farms and other producers stopped using Baytril before the July 2005 ban, an industry spokesman said alternative drugs are not as effective in dealing with respiratory illnesses in mass-produced poultry.

The ban is intended to stop the increase of drug-resistant strains of foodborne *Campylobacter. Campylobacter* infection causes abdominal symptoms and fever, and is one of the most common bacterial causes of diarrheal illness in the United States. According to the FDA, 20% of human *Campylobacter* infections involve the resistant strain.

## WHO Knows About Radon?

The WHO has launched the International Radon Project to educate the public about the hazards of this chemically inert, radioactive gas that occurs naturally in soils and rocks around the world. The project will include a database of average radon levels in member nations, radon action levels, and mitigation measures, among other information. The WHO has also published a new fact sheet on radon and cancer as part of the project.

Radon may cause 6–15% of lung cancer cases, and moderate exposure may increase the risk of lung cancer in smokers by 25 times. Radon exposure in homes varies according to a home’s location, ventilation, and presence of exterior cracks and openings.

## From Carpet to Kilowatts

Each year some 4.7 billion pounds of carpet are taken to U.S. dumps, taking up almost 1% of the country’s landfill space. Now Shaw Industries, the world’s largest carpet maker, has opened a $10 million power plant that is fueled by the 16,000 tons of scrap the company turns out annually as well as by 6,000 tons of sawdust produced by wood flooring manufacturing. The new plant powers one of the company’s main factories, and should save the company $2.5 million in fuel oil each year. The plant engineers say the process emits about the same amount of pollution as natural gas.

## Figures and Tables

**Figure:**
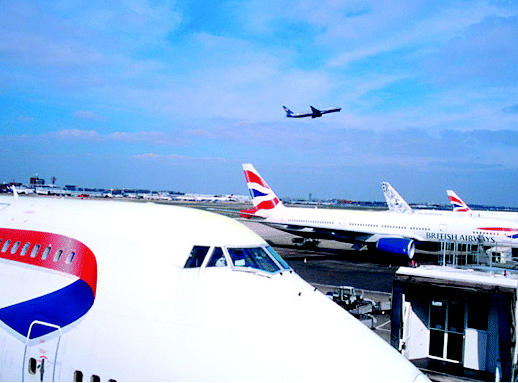


**Figure:**
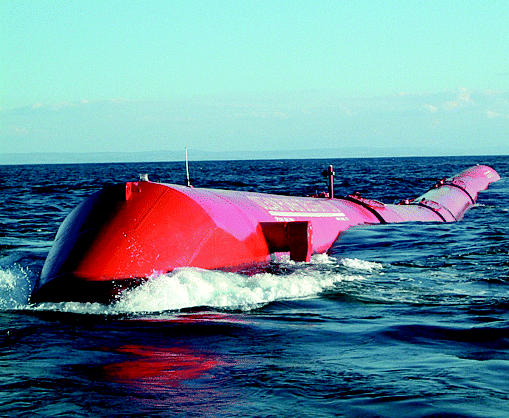


**Figure:**
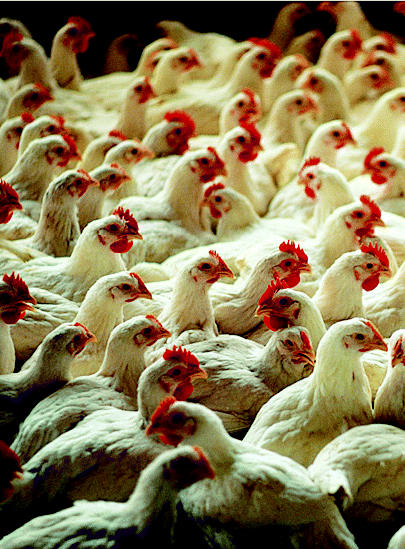


**Figure:**